# A common conformationally coupled ATPase mechanism for yeast and human cytoplasmic HSP90s

**DOI:** 10.1111/j.1742-4658.2008.06773.x

**Published:** 2009-01

**Authors:** Cara K Vaughan, Peter W Piper, Laurence H Pearl, Chrisostomos Prodromou

**Affiliations:** 1 Section of Structural Biology, The Institute of Cancer Research, Chester Beatty LaboratoriesLondon, UK; 2 Department of Molecular Biology and Biotechnology, The University of SheffieldUK

**Keywords:** ATPase activity, chaperone, heat shock protein, Hsp90, N-terminal dimerization

## Abstract

The conformationally coupled mechanism by which ATP is utilized by yeast Hsp90 is now well characterized. In contrast, ATP utilization by human Hsp90s is less well studied, and appears to operate differently. To resolve these conflicting models, we have conducted a side-by-side biochemical analysis in a series of mutant yeast and human Hsp90s that have been both mechanistically and structurally characterized with regard to the crystal structure of the yeast Hsp90 protein. We show that each monomer of the human Hsp90 dimer is mutually dependent on the other for ATPase activity. Fluorescence studies confirmed that the N-terminal domains of Hsp90β come into close association with each other. Mutations that directly affect the conformational dynamics of the ATP-lid segment had marked effects, with T31I (yeast T22I) and A116N (yeast A107N) stimulating, and T110I (yeast T101I) inhibiting, human and yeast ATPase activity to similar extents, showing that ATP-dependent lid closure is a key rate-determining step in both systems. Mutation of residues implicated in N-terminal dimerization of yeast Hsp90 (L15R and L18R in yeast, L24R and L27R in humans) significantly reduced the ATPase activity of yeast and human Hsp90s, showing that ATP-dependent association of the N-terminal domains in the Hsp90 dimer is also essential in both systems. Furthermore, cross-linking studies of the hyper-active yeast A107N and human A116N ATP-lid mutants showed enhanced dimerization, suggesting that N-terminal association is a direct consequence of ATP binding and lid closure in both systems.

How ATP is utilized by heat shock protein 90 (Hsp90) has been the subject of some controversy [1–3]. Subsequent structural, biochemical and cellular studies [4–7] demonstrated unequivocally that Hsp90 binds and hydrolyses ATP *in vitro*, and its ability to do so is essential to its role as a molecular chaperone *in vivo*.

Despite general acceptance of Hsp90 as an ATP-dependent molecular machine [8], the detailed mechanism by which Hsp90 hydrolyses ATP, and the coupling of this activity to conformational changes in the Hsp90 dimer, remain controversial. Studies of yeast Hsp90 have defined a coherent ATPase-coupled conformational cycle in which ATP binding and hydrolysis are linked to the opening and closing of a molecular clamp involving transient ATP-dependent association of the N-terminal domains in the Hsp90 dimer [9–12]. However, no cooperation between the N-terminal domains has been seen for human Hsp90, suggesting that they operate as independent units with no functional requirement for association [13].

While it is possible that yeast and human Hsp90s operate by different mechanisms, their high overall sequence identity (61.5%), their near-complete conservation of all residues involved in ATP transactions, and the fact that structural studies have shown that the yeast and human Hsp90α N-terminal domains are virtually identical make this unlikely [14,15]. In an attempt to resolve this issue, we have conducted a side-by-side biochemical analysis of a matched series of yeast and human Hsp90 proteins mutated at residues implicated in the ATP-coupled conformational mechanism deduced from the yeast studies, the mechanistic and structural consequences of which can be understood in the context of the crystal structure of full-length yeast Hsp90 [9]. These studies suggest an essentially identical set of conformational changes that accompany the ATPase cycle in both species.

## Results

### C-terminal truncation mutants of Hsp90β have a reduced ATPase activity

**Fig. 1:**
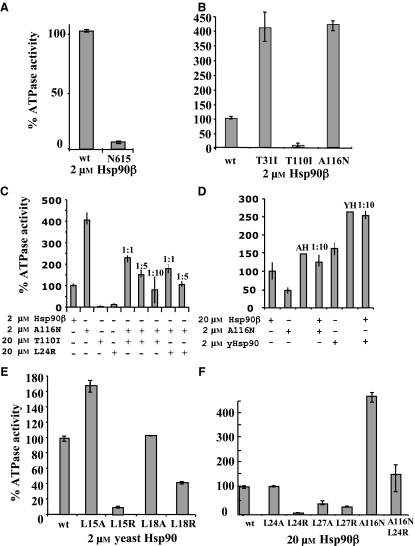
ATPase activity of yeast Hsp90 and human Hsp90β. The relative ATPase activities for (A) the C-terminal truncation mutant Hsp90β N615, (B) the Hsp90β mutations affecting ATP-lid conformation, (C) the A116N–T110I and A116N–L24R heterodimer experiments, (D) the Hsp90β–A116N and Hsp90β–yeast Hsp90 heterodimer experiments, (E) mutations directly affecting N-terminal dimerization of yeast Hsp90, and (F) mutations directly affecting N-terminal dimerization of human Hsp90β. The molar concentrations of the Hsp90 constructs used are shown. Assays were performed at 37 °C for 15–60 min. Geldanamycin at a concentration of 30–60 μm was used to obtain the baseline activity of contaminating background ATPase. For the Hsp90 heterodimer assays, 2 μm of A116N was used with increasing amounts of T110I, L24R, Hsp90β and yeast Hsp90, and the relative molar concentration used is shown above the corresponding activity. All assays were performed at least three times, and the *k*_cat_ values for yeast and human Hsp90 were 0.91 and 0.056/min, respectively, at 37 °C.

We have previously shown that removal of the C-terminal dimerization domain of yeast Hsp90 greatly reduces its ATPase activity [10]. Although the truncated protein contains all the catalytic machinery necessary to hydrolyse ATP, the absence of the constitutive dimerization interface makes ATP-dependent N-terminal association less efficient, showing that N-terminal association is required for ATP turnover. When we measured the ATPase activity of a similarly C-terminally truncated human Hsp90β construct (residues 1–615), we found that its ATPase activity was also drastically reduced compared to the wild-type (Fig. 1A), consistent with a previous study [13]. Thus C-terminal truncation in human Hsp90β and in yeast Hsp90 drastically reduces both their ATPase activities, suggesting that it affects a common mechanistic feature, and this is fully consistent with the assumption that N-terminal dimerization and therefore the ATPase activity of Hsp90β are assisted by physical association of the two molecules within each Hsp90 dimer at their C-termini.

### Mutations affecting ATP-lid closure in yeast and human Hsp90 suggest a common mechanism for the hydrolysis of ATP

Work using yeast Hsp90 has shown that the ATPase activity is dependent on transient association of the N-terminal nucleotide-binding domains within the Hsp90 dimer [10]. Binding of ATP to the yeast Hsp90 N-terminal domain promotes remodelling and closure of a segment of polypeptide (residues 94–123 in yeast, equivalent to 103–132 in human Hsp90β) that forms a ‘lid’ over the nucleotide-binding site [9]. Closure of the ATP lid in each Hsp90 monomer exposes hydrophobic surfaces that can interact with each other within the dimer and thus stabilize N-terminal association.

**Fig. 2:**
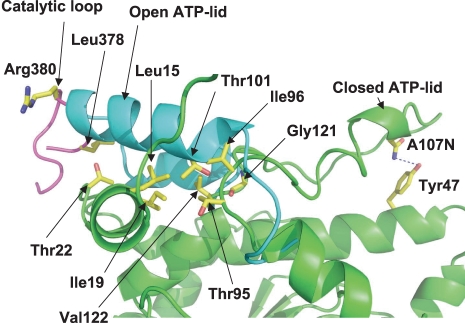
Molecular interactions of yeast ATP-lid mutations. The ATP lid in its closed state brings the A107N mutation in close proximity to Tyr47, with which it interacts. This stabilizes the ATP lid in the closed state. In contrast, Thr101 is on the underside of the ATP lid in its open state (shown in turquoise). An isoleucine at this position increases hydrophobic interactions with similar residues in its vicinity. Thr22 in the closed state contacts the catalytic loop (shown in magenta), interacting with Leu378 of the neighbouring middle domain of the Hsp90 dimer. An isoleucine at this position would increase the hydrophobic nature of the interaction with Leu378 and would favour N-terminal dimerization.

We have previously characterized a series of mutations of yeast Hsp90 that alter ATPase activity by changing the dynamics of the coupled lid-closure and N-terminal-association processes [10] (Fig. 2). The A107N mutation promotes N-terminal association and greatly enhances ATPase activity by stabilizing the ATP lid in the closed conformation via formation of an additional polar interaction with Tyr47. The T22I mutation has a similar effect on N-terminal dimerization and ATP hydrolysis by increasing the hydrophobicity of the N-terminal dimerization interface (Fig. 2). In contrast, the increased hydrophobicity of the underside of the ATP lid resulting from the T101I mutation stabilizes the lid in its open state, and therefore decreases ATPase activity. We investigated the effect of the equivalent mutations in the N-terminal domain of Hsp90β on its ATPase activity. Consistent with the previous observations in yeast Hsp90, the T31I (yeast T22I) and A116N (yeast A107N) mutations both enhanced the ATPase activity of Hsp90β, while the T110I mutation (yeast T101I) drastically reduced it (Fig. 1D). The equivalent mutations in human and yeast Hsp90 therefore affect ATPase activity in the same way, suggesting that they affect mechanistic features common to both, namely ATP-lid closure and N-terminal association.

### The monomers of the Hsp90 dimer are mutually dependent on each other for ATPase activity

The results so far are consistent with the suggestion that human Hsp90 undergoes an ATP-coupled conformational change that leads to dimerization of its N-terminal domains. However, a direct dependence of one monomer of Hsp90 on the other monomer has not yet been demonstrated. In order to show this, we formed heterodimers (A116N–T110I and A116N–L24R), as previously described [16], by mixing 2 μm human Hsp90β carrying the A116N mutation with up to a tenfold molar excess of the T110I mutant and a fivefold molar excess of the L24R mutant. Results presented below suggest that the L24R mutant prevents N-terminal dimerization. Consequently, with increasing molar ratios of the T110I and L24R mutants, relative to the fixed amount of A116N, we expect the concentration of the A116N–T110I and A116N–L24R heterodimers to increase relative to free A116N homodimer. Thus the ATPase activity that is detected is increasingly due to heterodimers rather than free homodimers of A116N (the free homodimers of L24R and T110I also contribute to the overall activity, but their contribution is low due to their inherently low ATPase activity). If the activity of each monomer is independent of its dimeric partner, the activity of A116N would be unaffected by the T110I or L24R mutation in the adjacent monomer. Consequently, we would detect an activity that is the sum for the A116N and T110I or L24R activities. However, if the activity of A116N were dependent on its dimeric partner, we would see a decrease in the ability of the A116N monomer to hydrolyse ATP, and the total activity detected would decrease. The results in Fig. 1C show that the T110I and L24R mutations did indeed decrease the overall ATPase activity detected, which suggests that they affect the ability of A116N to hydrolyse ATP. To test the possibility that the L24R and T110I mutants affect the ATPase by some non-specific mechanism, we also added a tenfold molar excess of human Hsp90β to 2 μm A116N. As this experiment utilizes two molecules that are both substantially active, the effect on the overall ATPase activity should be to approach that of Hsp90β, since it is not only in excess, but less active and would limit the ATPase activity of the more active A116N, as seen with the A116N–L24R and A116N–T110I heterodimers. We also added a tenfold molar excess of Hsp90β to 2 μm yeast Hsp90, which are unlikely to heterodimerize because their C-terminal dimerization interfaces are not completely identical. Consequently, we expect to see an activity that is the sum of the two separate activities (Hsp90β + yeast Hsp90). The results shown in Fig. 1D are consistent with this. The ATPase activity of the A116N/Hsp90β mixture was slightly higher than that of Hsp90β alone, but less than the combined sum of the A116N and Hsp90β activities. Furthermore, yeast Hsp90 had no effect on the ATPase activity of human Hsp90, suggesting that inhibition of A116N is unlikely to be due to non-specific effects on the A116N protein (Fig. 1D). In conclusion, the results suggest that the monomers of the Hsp90 dimer are mutually dependent on each other for the hydrolysis of ATP. Such cooperation between the monomers of human Hsp90 is consistent with association of the N-terminal domains, as seen with yeast Hsp90.

### AMPPNP promotes N-terminal dimerization

**Fig. 3:**
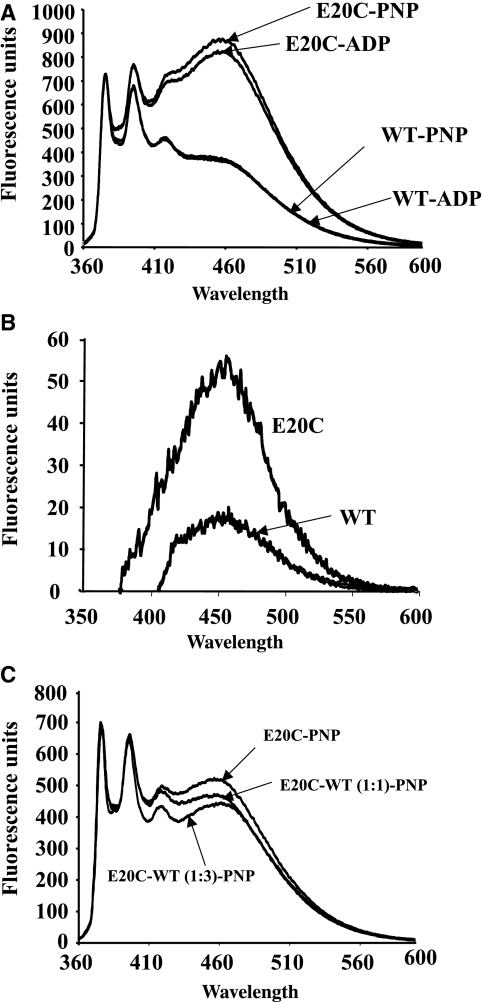
N-terminal interactions of pyrene-labelled cysteine mutants. (A) Specific excimer fluorescence signals normalized for the pyrene-labelled E20C-A116N-C366A (E20C) protein and pyrene-treated control protein A116N-C366A (WT) in the presence of either AMPPNP (PNP) or ADP. Spectra were obtained for three separate labelling reactions, and the results of a typical experiment are shown. The results show that the WT signal is similar in the presence of either AMPPNP or ADP, whereas the mutant specifically labelled at position E20C shows an increase in excimer fluorescence in the presence of AMPPNP over that with ADP. (B) Specific excimer fluorescence signals for the E20C mutant and the control protein (WT) in the presence of AMPPNP, corrected for the signal in the presence of ADP. The traces are the mean of three separate experiments. The E20C mutant displays enhanced excimer fluorescence relative to the control protein. (C) Specific excimer formation of the labelled protein E20C in the presence of increasing amounts of WT control protein. The WT protein specifically reduces excimer fluorescence, indicating that it forms heterodimers with the E20C protein and thus reduces interactions between the pyrene-labelled E20Cs of two monomers upon AMPPNP binding. Excimer fluorescence occurs in the wavelength of 450–500 nm.

The ATPase activity results of the heterodimeric A116N–T110I and A116N–L24R experiments suggest that the N-terminal domains are dependent on each other for their ATPase activity. We used fluorescence resonance energy transfer (FRET) to investigate the conformation of Hsp90 as previously described for the yeast protein [10]. When excited and in close contact, pyrene molecules display a marked alteration in fluorescence emission at 450–500 nm (excimer formation). A structural analysis revealed that human Hsp90β contains six cysteine residues, four of which are buried and cannot be labelled without denaturing the protein. The fifth is present within the C-terminal domain, but in a position that would prevent pyrene from interacting with a second pyrene at the equivalent position on the adjacent monomer. The sixth cysteine residue (Cys366) is present within the middle domain. Potentially, this residue would allow excimer formation if labelled with pyrene. We therefore mutated this surface cysteine to alanine, and introduced a new cysteine residue by mutating Glu20 (E11C in yeast Hsp90) in the A116N background. The A116N mutant was chosen as this would maximize the excimer signal that could be generated, which we expected to be low due to the lower inherent ATPase activity of Hsp90β. The fluorescent emission spectrum of labelled E20C-A116N-C336A, either in the presence of ADP or AMPPNP, showed significant excimer formation compared to control protein (A116N-C336A) subjected to the pyrene modification procedure (Fig. 3A), as previously seen with E11C pyrene-labelled yeast Hsp90. However, in contrast to the control C366A-A116N protein, the excimer signal for the E20C-C336A-A116N pyrene-labelled protein was enhanced in the presence of AMPPNP compared to ADP (Fig. 3B), thus confirming that AMPPNP promotes a closer association of the N-terminal domains. Subsequently, we demonstrated that heterodimer formation between the pyrene-labelled E20C-C366A-A116N mutant protein and control protein (C366A-A116N) in the presence of AMPPNP resulted in a decrease in excimer fluorescence (Fig. 3C). Thus, it appears that heterodimer formation decreases interactions between pyrene-labelled E20Cs on the two monomers, as the control protein (C366A-A116N) lacks one of the specific pyrene labels at position 20. The results, as a whole, suggest that, as for the yeast protein, human Hsp90β also undergoes N-terminal dimerization.

### N-terminal dimerization is essential for ATPase activity in yeast and human Hsp90

**Fig. 4:**
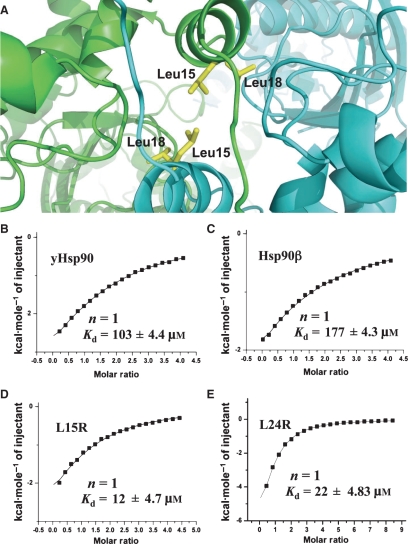
Part of the N-terminal dimerization interface of Hsp90 and binding of AMPPNP to N-terminal dimerization mutants. (A) The two chains of the Hsp90 dimer are shown in green and turquoise. Two hydrophobic residues (Leu15 and Leu18) were identified as potential sites for mutation in order to disrupt N-terminal association. Mutations to alanine can be easily accommodated at these sites, whereas a change to arginine is likely to be disruptive. (B–E) AMPPNP binding to yeast Hsp90 (B), human Hsp90β (C), the yeast L15R mutant (D) and the human L24R mutant (E) by isothermal titration calorimetry.

The ATP-lid mutations and the mutual dependency of the monomers of dimeric Hsp90β for ATPase activity suggest that the human protein undergoes N-terminal dimerization, in common with yeast Hsp90. Mutations in yeast Hsp90 that directly inhibit N-terminal dimerization, and therefore ATPase activity, have not been previously described. Analysis of the structure of the yeast Hsp90–AMPPNP–Sba1 complex [9] suggested that Leu15 and Leu18, which play no direct catalytic role in the hydrolysis of ATP, are important for N-terminal dimerization, as they form part of the hydrophobic core for this interaction (Fig. 4A). We therefore mutated Leu15 and Leu18 to either alanine or arginine, and determined the effects of these mutations on ATPase activity of Hsp90, which is a measure of N-terminal dimerization. We found that the L15A and L18A mutations, which could be accommodated structurally, had no inhibitory effect on the ATPase activity of yeast Hsp90 (157 and 103% activity, respectively, relative to wild-type yeast Hsp90; Fig. 1E). In fact, the L15A mutation activated the ATPase activity of yeast Hsp90. However, L18R, and in particular the L15R mutation, which prevent formation of the dimer interface, significantly reduced ATPase activity (42 and 9.5% activity, respectively, relative to wild-type yeast Hsp90; Fig. 1E). This suggests that N-terminal dimerization is compromised by both the L15R and L18R mutations, and confirms that the ATPase activity of yeast Hsp90 is dependent on close association of the N-terminal domains. Having identified mutations in yeast Hsp90 that directly inhibit N-terminal dimerization, we created the equivalent mutant forms of Hsp90β and evaluated their ability to N-terminally dimerize by determining their ATPase activity. We found that the ATPase activity of L24A was almost identical to that of wild-type Hsp90β (101% activity relative to wild-type Hsp90β; Fig. 1F), whereas the L27A mutation showed a somewhat reduced activity because Alanine at this position disfavors dimerization (30% activity relative to wild-type Hsp90β; Fig. 1F). Consistent with N-terminal dimerization, the L24R and L27R mutations also showed a reduction in ATPase activity (6.2 and 17% activity, respectively, relative to wild-type Hsp90β; Fig. 1F). Although it is possible to envisage other means by which these mutants might affect the ATPase activity of human Hsp90, these results are nonetheless consistent with the idea that the ATPase activity of human Hsp90β, like that of yeast Hsp90, is dependent on N-terminal dimerization. It is noted that the L15A mutation activated the ATPase activity of yeast Hsp90, whereas the equivalent mutation (L24A) in Hsp90β had very little effect. Similarly, the L18A mutation had a less severe effect on the ATPase activity of yeast Hsp90 than the equivalent (L27A) mutation of Hsp90β. These slight differences are to be expected in such a complex system, and probably reflect differences in the overall dynamics and stability of the dimerization process. Nonetheless, we have identified mutations in yeast (L15R and L18R) that are consistent with the disruption of N-terminal dimerization. Furthermore, when the equivalent mutations are made in Hsp90β (L24R and L27R), its ATPase activity is also disrupted, suggesting that it too undergoes N-terminal dimerization. We next tested the binding of AMPPNP to the L15R and L24R mutant Hsp90s to exclude the possibility that the mutations inhibited the binding of ATP. We found that neither mutation inhibited binding of the non-hydrolysable ATP analogue, AMPPNP (Fig. 4B–E). Instead, we noticed an increase in affinity for AMPPNP (yeast Hsp90, *K*_d_ = 103 ± 4.4 μm; L15R, *K*_d_ = 12 ± 4.7 μm; human Hsp90β, *K*_d_ = 177 ± 3.3μm; L24R, *K*_d_ = 22.0 ± 0.83 μm) that probably reflects a more accessible active site due to the mutations shifting the equilibrium away from the closed state.

### AMPPNP promotes N-terminal dimerization of Hsp90β

**Fig. 5:**
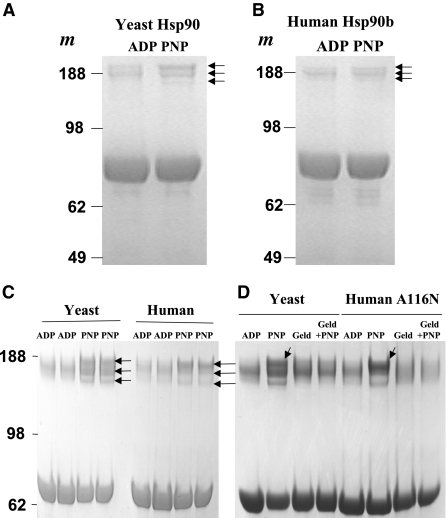
Cross-linking reactions with yeast Hsp90 and Hsp90β. Cross-linking with DMS in the presence of ADP or AMPPNP for (A) yeast Hsp90 and (B) human Hsp90β for 15 min, or (C) yeast and human Hsp90 for 60 min. (D) Inhibition of cross-linking of yeast and human Hsp90s by geldanamycin. All cross-linking assays were performed in triplicate, and typical results are shown. Proteins for cross-linking were diluted to a final concentration of 0.25–0.5 mg·mL^−1^ in 100 mm Hepes, pH 8.5, 150 mm KCl and 5 mm MgCl_2_, with either 5 mm ADP-Mg^2+^ or AMPPNP-Mg^2^. For yeast Hsp90 and Hsp90β reactions, 7.5 and 3.75 m excesses of DMS, respectively, were used relative to the molar concentration of Hsp90 lysine amino acid residues. Cross-linked dimer formation is enhanced in the presence of AMPPNP for both yeast and human Hsp90. In particular, cross-linking in the presence of AMPPNP generates a slowly migrating cross-linked species (shown by the topmost arrow) that is most likely formed from N-terminal dimers of the proteins. However, the amino acid residues that were cross-linked in the ADP experiment are close enough in the AMPPNP state to be subject to cross-linking. Formation of this slowly migrating band (shown by the slanted arrow) is inhibited by geldanamycin, even when AMPPNP is present, producing a cross-linking pattern similar to that with ADP.

The results so far suggest that human Hsp90 undergoes an ATP-coupled conformational change that leads to dimerization of its N-terminal domains. We investigated whether the non-hydrolysable ATP analogue, AMPPNP, could drive N-terminal dimerization of the yeast and human Hsp90s by using the cross-linking reagent dimethyl suberimidate (DMS) to capture the transient N-terminally dimerized state. The results in Fig. 5A–Dand Fig. S1 show that AMPPNP promotes the enhancement of cross-linked dimers, relative to ADP, thus suggesting that AMPPNP binding leads to N-terminal dimerization in both the human and yeast proteins. It should be noted that, as Hsp90 is inherently dimeric, both ADP and AMPPNP allow formation of cross-linked dimers; however, there is a consistent enhancement of cross-linked dimer formation in the presence of AMPPNP. In the presence of AMPPNP, the band with the highest molecular mass, representing cross-linked dimer species, was more abundant than in the presence of ADP for both the yeast and human Hsp90s (Fig. 5A–D and Fig. S1). This indicates that the ADP– and AMPPNP–Hsp90 complexes are structurally distinct. Thus, it appears that high molecular mass species are preferentially formed in the preference of AMPPNP (Fig. 5C and Fig. S1), which promotes the N-terminally dimerized state, while the faster running bands result mainly from the inherently dimerized open state. However, it should be noted that the cross-links that are formed in the presence of ADP are also formed in the closed state with AMPPNP. Thus, the amino acid residues that were cross-linked in the ADP experiment are close enough in the AMPPNP state to be subject to cross-linking. Whether the slowly migrating band results from a single cross-link and the location of such cross-links is unknown. N-terminal dimerization would result in not only the N-terminal domains associating, but also in the middle domains coming into closer contact. Thus potentially many cross-links are possible, and their location need not necessarily be limited to the N-terminal domains. However, in order to confirm that AMPPNP drives N-terminal dimerization and allows formation of the slowly migrating band, we used geldanamycin, an Hsp90 inhibitor known to bind to the ATP-binding pocket of Hsp90 and prevent N-terminal dimerization. Using the A116N mutant of Hsp90β, which shows enhanced formation of this slowly migrating band (see Results below), we demonstrated that geldanamycin, and indeed ADP, both severely inhibit formation of the slowest migrating cross-linked species relative to AMPPNP, in common with the results for yeast Hsp90 (Fig. 5D and Fig. S1).

### ATP-lid and N-terminal dimerization mutations modulate the degree of cross-linking of human Hsp90

**Fig. 6:**
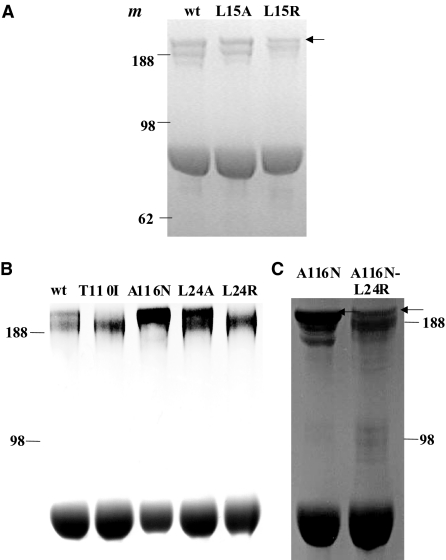
Cross-linking reactions with mutations of yeast Hsp90 and Hsp90β. Cross-linking with DMS in the presence of AMPPNP for (A) yeast Hsp90 and the L15A and L15R mutations for 15 min, or (B) human Hsp90β and the T110I, A116N, L24A and L24R mutations for 60 min, or (C) the L24R-A116N mutation of Hsp90β for 60 min. The arrow shows the position of the slowly migrating cross-linked species, which are favourably formed with the A116N mutant. Proteins for cross-linking were diluted to a final concentration of 0.25–0.5 mg·mL^−1^ in 100 mm Hepes, pH 8.5, 150 mm KCl and 5 mm MgCl_2_, with either 5 mm ADP-Mg^2+^ or AMPPNP-Mg^2^. For yeast Hsp90 and Hsp90β reactions, 7.5 and 3.75 m excesses of DMS, respectively, were used relative to the molar concentration of Hsp90 lysine amino acid residues. The N-terminal dimerization mutations, L15R (yeast) and L24R (human), as well as T110I (T101I in yeast) reduce formation of the slowly migrating cross-linked species (shown by the arrow), which is consistent with the ATP-lid model. All cross-linking assays were performed in triplicate, and typical results are shown.

Mutations that affect either the conformation of the ATP lid or block dimerization directly are expected to affect the degree of cross-linking by inhibiting AMPPNP-induced N-terminal dimerization. We have previously shown that the ATP-lid mutation of yeast Hsp90, T101I, inhibits N-terminal dimerization and therefore the formation of cross-linked dimers, whereas A107N promotes dimerization and cross-linking [10]. The results in Fig. 6A and Fig. S2 show that the yeast Hsp90 mutation L15R inhibits cross-linking, as expected, and suggest that this mutation acts by directly preventing N-terminal dimerization. Likewise, the equivalent mutation, L24R, as well as the T110I mutation (yeast T101I), of human Hsp90 inhibited cross-linking, while the A116N mutation (yeast A107N) promoted it, as expected (Fig. 6B and Fig. S1). These differences in cross-linking are particularly apparent by the extent of formation of the slowest migrating cross-linked species, which was inhibited by ADP and geldanamycin (or enhanced by AMPPNP) (Fig. 5A–D and Fig. S1). This species is most likely formed as a result of cross-linking of the protein in an N-terminally dimerized state. Thus, for mutations inhibiting N-terminal dimerization, the bulk of the cross-linked products appear to migrate at a lower position than the products from mutations that promote N-terminal dimerization (compare A116N, T110I and L24R in Fig. 6B and Fig. S2). However, in order to verify this result, we decided to investigate the ability of the L24R mutation, which appears to prevent N-terminal dimerization, to disrupt formation of the slowly migrating cross-linked product observed for the A116N mutant. The results in Fig. 6C and Fig. S2 show that the combination of the L24R and A116N mutations clearly disrupts formation of this cross-linked product and validates the conclusions drawn from the results shown in Fig. 6B and Fig. S2. This is also consistent with the dependency of the ATPase activity of A116N on the L24R mutation in heterodimer experiments. Collectively, the results show that the effects caused by these mutations on ATPase activity and N-terminal dimerization are the same for both the human and yeast Hsp90s, and support the view that human Hsp90 ATPase activity is also subject to ATP-coupled N-terminal dimerization.

## Discussion

In yeast, the rate-limiting step for ATP turnover appears to involve a complex series of structural rearrangements that lead to formation of a catalytically active unit that is able to hydrolyse ATP [11], and mutations (T22I, T101I and A107N) that affect formation of the catalytically active state have been described [10]. In this study, we show that a C-terminally truncated Hsp90β, which contains all the catalytic machinery required for hydrolysis of ATP, and mutants with alterations that inhibit conformational changes in the ATP lid or prevent dimerization directly all show low ATPase activity, as expected if the mode for hydrolysis of ATP is based on the known mechanism for yeast Hsp90 [9]. These same mutations also inhibit the formation of cross-linked products that are favourably formed in the presence of AMPPNP, but whose formation is inhibited by ADP and geldanamycin. The results collectively suggest a common mechanism for hydrolysis of ATP by yeast and human Hsp90. Furthermore, our results suggest that the ATPase activity of each Hsp90β monomer is dependent on the adjacent monomer. This implies that each monomer must interact with its partner monomer, and is strong evidence for N-terminal dimerization in Hsp90β. This was further supported by fluorescence studies showing that AMPPNP promotes a closer association of the N-terminal domains of human Hsp90 than ADP. Consequently, our results, together with the highly conserved amino acid sequence similarity between the yeast and human Hsp90β N-terminal domains, lead us to conclude that hydrolysis of ATP by yeast and human Hsp90 shares a common mechanism.

Although both yeast and human Hsp90 appear to utilize ATP via the same mechanism, their activity is not identical. In this study, the *k*_cat_ values were estimated as 0.91 and 0.056/min for yeast and human Hsp90β, respectively, at 37 °C. The lower activity seen for the human protein suggests that N-terminal dimerization, the rate-limiting step of ATP hydrolysis, is slower in human Hsp90β. Thus a larger energy barrier exists for formation of a stable N-terminally dimerized state in Hsp90β. It would appear that binding of ATP to Hsp90, although promoting N-terminal dimerization, is not the only determining factor that governs the stability of the closed state. This is not surprising as formation of the catalytic state is dependent on a number of conformational changes within Hsp90, such as release of the catalytic loop from its middle domain [9,17]. These additional structural changes allow the ATPase activity of Hsp90 to be regulated not only by the availability of ATP, but by co-chaperones that affect parts of Hsp90 that are critical for formation of a catalytic state. This has been clearly demonstrated in structural studies that have shown how the co-chaperones Aha1 and p23/Sba1 regulate the ATPase activity of Hsp90 [9,17]. Indeed, by such mechanisms, the ATPase activity of Hsp90 may also be influenced by the nature of the client protein in the Hsp90 complex. In fact, such co-chaperone interactions, which appear to be similar for both the yeast and human Hsp90 proteins, provide further evidence in support that the human and yeast mechanisms are similar. For example, we have shown that yeast Aha1 can stimulate the ATPase activity of both yeast and human Hsp90s [11,18,19], while Sba1, which is known to bind to the closed state of yeast Hsp90, inhibits the ATPase activity of the human and yeast proteins [9,19,20]. Furthermore, Sti1 can inhibit the ATPase activity of both yeast and human Hsp90s [11,18,19], and yeast and human Cdc37 inhibit the ATPase activity of yeast Hsp90 [9–12].

During the course of this work, it was shown that the ATPase activity detected in human Hsp90β heterodimer experiments using one intact monomer and another monomer lacking the N-terminal domain was reduced [21]. This suggested that the monomers of Hsp90β are dependent on each other for the hydrolysis of ATP. This is in full agreement with our experiments in which we produced subtle mutations in residues of the N-terminal domain of human Hsp90β that are known to be involved in N-terminal dimerization of yeast Hsp90, and performed heterodimer experiments with these mutants (T110I or L24R) and wild-type Hsp90β that resulted in reduced ATPase activity. Richter *et al.* [21] also showed that bound ATP remains solvent-accessible, and concluded either that the closed state does not exist or that it is less populated relative to the open state. Our results suggest that a closed state is formed, but is either less stable or is formed less readily than observed for the yeast protein. This difference in the ability of human and yeast to form a catalytically active closed state is reflected in their ATPase activity, which is 16 times lower for Hsp90β (*k*_cat_ for yeast = 0.91/min, *k*_cat_ for Hsp90β = 0.056/min at 37 °C).

During the preparation of this manuscript, evidence suggesting that constitutively expressed yeast Hsc90 hydrolyses ATP in an independent manner, not utilizing N-terminal dimerization, was published [22]. In these experiments, an inactive E33A mutant was mixed with and had no effect on wild-type Hsc90 ATPase activity. It was therefore concluded that the two halves of the dimer act independently to hydrolyse ATP. However, as the E33A mutant is fully able to bind ATP, it might still be subject to ATP-lid closure, thus providing a platform with which the adjacent active monomer can N-terminally dimerize. Following hydrolysis by the wild-type monomer, it might then disengage from the closed E33A monomer and enter a second round of ATP hydrolysis. In contrast, the L24R mutant in our model prevents N-terminal dimerization and reduces the ATPase activity of the A116N mutant, thus suggesting a dependent mechanism based on N-terminal dimerization.

Whether the 94 kDa glucose-regulated Hsp90 of the endoplasmic reticulum, Grp94, can hydrolyze ATP has also been very controversial. Amino acid sequence analysis suggests that Grp94 should be able to hydrolyse ATP, but this activity has not been identified. However, ATPase activity for canine Grp94 has recently been detected, although the activities reported, *k*_cat_ *=*0.02/min at 37 °C and 0.36/min at 25 °C, vary [23,24]. ATPase activity has also been reported for the mitochondrial Hsp90 TRAP1 (*k*_cat_ *=*0.1/min at 30 °C and 0.16/min at 25 °C [25,26]), as well for *Escherichia coli* high temperature protein G (HTPG) (*k*_cat_ *=*0.16/min at 37 °C [6]). In conclusion, our results suggest that eukaryotic Hsp90s, and perhaps all types of Hsp90, utilize ATP via the same structural mechanism, and that differences in the level of activity are due to varying stability of the N-terminally dimerized state induced through ATP binding.

## Experimental procedures

### Protein production and Hsp90 ATPase activity assays

Expression and purification of the His-tagged yeast Hsp90 and human Hsp90β, including mutant forms, was performed as previously described [11,18,19]. C-terminally truncated His-PreScission-Hsp90β (N615, amino acid residues 1–615) was cloned into pTwoE (pET-17b engineered to contain a His tag and a PreScission cleavage site; personal communication, A. W. Oliver, Institute of Cancer Research, London, UK) as an *Nhe*I–*Spe*I DNA fragment, and expressed and purified as for the Hsp90β protein. Purified proteins were dialysed against 20 mm Tris/HCl, pH 7.5, containing 1 mm EDTA and 1 mm dithiothreitol, and then concentrated using Vivaspin concentrators (Sartorius, Epsom, UK) with an appropriate molecular mass cut-off. The ATPase assays were performed as previously described [18,19] using 2 μm yeast or 2–20 μm human Hsp90 depending on activity. All samples were assayed at least three times, and some up to 12 times. For Hsp90 heterodimer formation, the Hsp90 isoforms were mixed in a ratio of 1 : 1 (2 μm A116N:2 μm T110I or L24R), 1 : 5 (2 μm A116N:10 μm T110I or L24R) or 1 : 10 (2 μm A116N:20 μm T110I; 2 μm A116N:20 μm Hsp90β and 2 μm A116N:20 μm yeast Hsp90), and incubated for 60 min prior to the assay. For heterodimer experiments, the total activity for each Hsp90 construct was determined separately and then in combination. The *k*_cat_ values for the yeast, human Hsp90β and A116N mutant proteins were 0.91, 0.056 and 0.24/min at 37 °C, respectively. Non-specific ATPase activity was determined by the addition of 30–60 μm geldanamycin.

### Mutagenesis

Single amino acid changes in yeast Hsp90 and human Hsp90β were generated using the QuickChange mutagenesis system (Stratagene, La Jolla, CA, USA) as previously described [10]. The following mutations were introduced: L15A, L15R, L18A and L18R for yeast Hsp90, and T31I, T110I, A116N, L24A, L24R, L27A, L27R, L24R-A116N, A116N-C366A and E20C-C366A-A116N for human Hsp90β. Mutations were confirmed by dye-terminator sequencing.

### Cross-linking

Proteins for cross-linking were diluted to a final concentration of 0.25–0.5 mg·mL^−1^ in 100 mm Hepes, pH 8.5, 150 mm KCl and 5 mm MgCl_2_. Either 5 mm ADP-Mg^2+^ or 5 mm AMPPNP-Mg^2+^ was added to the diluted protein. Cross-linking reactions were then carried out as described previously [10]. For yeast Hsp90 and Hsp90β reactions, 7.5 and 3.75 molar excesses of DMS, respectively, were used relative to the molar concentration of Hsp90 lysine amino acid residues. Reactions of 20 μL were incubated for either 15 or 60 min at room temperature. Reactions were stopped by the addition of 1 μL Tris, pH 6.8, and SDS loading buffer. Samples were analysed by 4–12% SDS–PAGE and stained with Coomassie blue.

### Fluorescent labelling and excimer formation

Fluorescent labelling was carried out as described previously [10]. Briefly, proteins for labelling were desalted in 100 mm Hepes, pH 8.0, containing 150 mm KCl. Triplicate samples of each protein were then diluted to 10 mg·mL^−1^, and the non-thiol reducing agent Tris(2-carboxyethyl)-phosphine was added to a final concentration of 10 mm. Subsequently, 10 mm*N-*(1-pyrene)-maleimide (in 100% dimethylformamide; Sigma-Aldrich, Poole, UK) was added, and the reaction was incubated at room temperature for 60 min. The reaction was then desalted twice into 20 mm Tris, pH 7.5, containing 1 mm EDTA. Fluorescence measurements were carried out at 10 μm protein concentration in 20 mm Tris, pH 7.4, 12 mm MgCl_2_, 150 mm KCl and either 10 mm ADP or AMPPNP. For heterodimer experiments, 5 μm of pyrene-labelled E20C-C366A-A116N mutant was mixed with control protein (C366A-A116N, pyrene treated but not labelled at position 20) in ratios of 1 : 1 and 1 : 3, respectively. Labelled proteins were excited at 341 nm and emission spectra obtained from 220 to 600 nm using a Cray Eclipse fluorescent spectrophotometer (Varian, Inc., Palo Alto, CA, USA). Normalized spectra of ADP-containing experiments were subtracted from normalized spectra from AMPPNP-containing experiments, and the resultant data were plotted.

### Isothermal titration calorimetry and *K*_d_ determinations

The heat of interaction was measured on an MSC system (Microcal Europe, Milton Keynes, UK), with a cell volume of 1.458 mL, under the same buffer conditions (20 mm Tris, pH 8.0, containing 1 mm EDTA and 5 mm NaCl) at 30 °C. Twenty 13.5 μL aliquots of 1 mm AMPPNP were injected into 50 μm of human or yeast Hsp90 or the L15R mutant or 24.3 μm L24R mutant. Heats of dilution were determined in a separate experiment by diluting protein into buffer, and the corrected data were fitted using a non-linear least-squares curve-fitting algorithm (Microcal Origin) with three floating variables: stoichiometry (fixed at 1.0), binding constant and change in enthalpy of interaction.

## Abbreviations

AMPPNP or PNP: adenosine 5′-(β,γ-imino)-triphospahte
DMS: dimethyl suberimidate
FRET: fluorescence resonance energy transfer
Grp94: 94 kDa glucose-regulated protein
Hsp90: heat shock protein 90

## Supporting information

The following supplementary material is available:

**Fig. S1.** Densitometry scans of the results shown in Fig. 5.

**Fig. S2.** Densitometry scans of the results shown in Fig. 6.

This supplementary material can be found in the online version of this article.
