# Supplementary material for: A common conformationally coupled ATPase mechanism for yeast and human cytoplasmic HSP90s
Source: FEBS J. 2009 Jan;276(1):199–209. doi: 10.1111/j.1742-4658.2008.06773.x (PMC2702006; doi:10.1111/j.1742-4658.2008.06773.x)
Supplement: Supplementary file 1 [file ejb0276-0199-SD1.pdf]

doi:10.1111/j.1742-4658.2008.06773.x

Supporting information

Fig S1. Densitometry scans of the results shown in Figure 5. Gels were scanned and were necessary normalized against total intensity. The peak representing the slowest migrating band for the yeast Hsp90 is shown by the black arrow.

Fig S2. Densitometry scans of the results shown in Figure 6. Gels were scanned and were necessary normalized against total intensity. The peak representing the slowest migrating band for the yeast Hsp90 is shown by the black arrow.

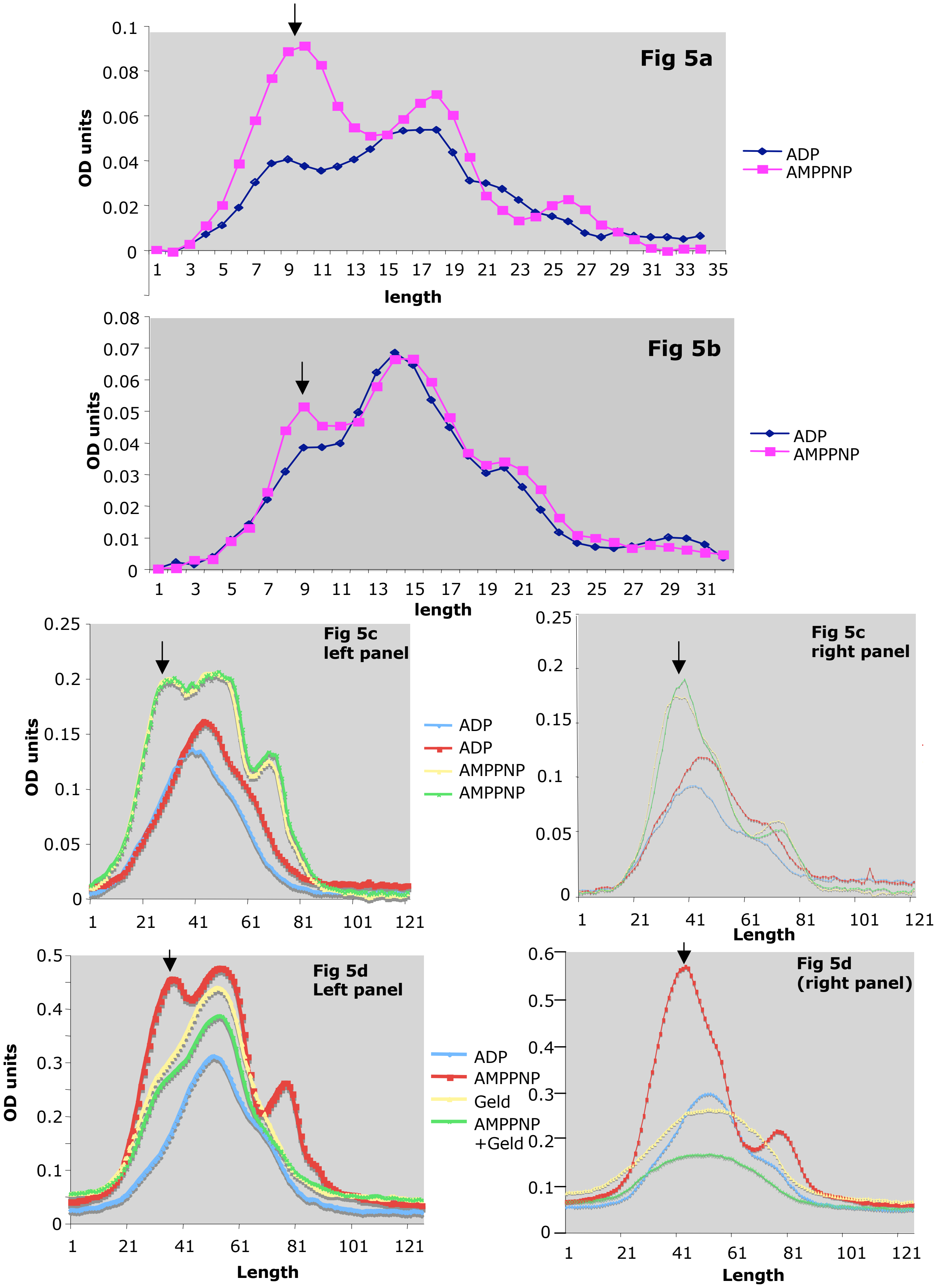

Supplementary figure 1. Gels were scanned and were necessary normalized against total intensity. The peak representing the slowest migrating band for the yeast Hsp90 is shown by the black arrow.

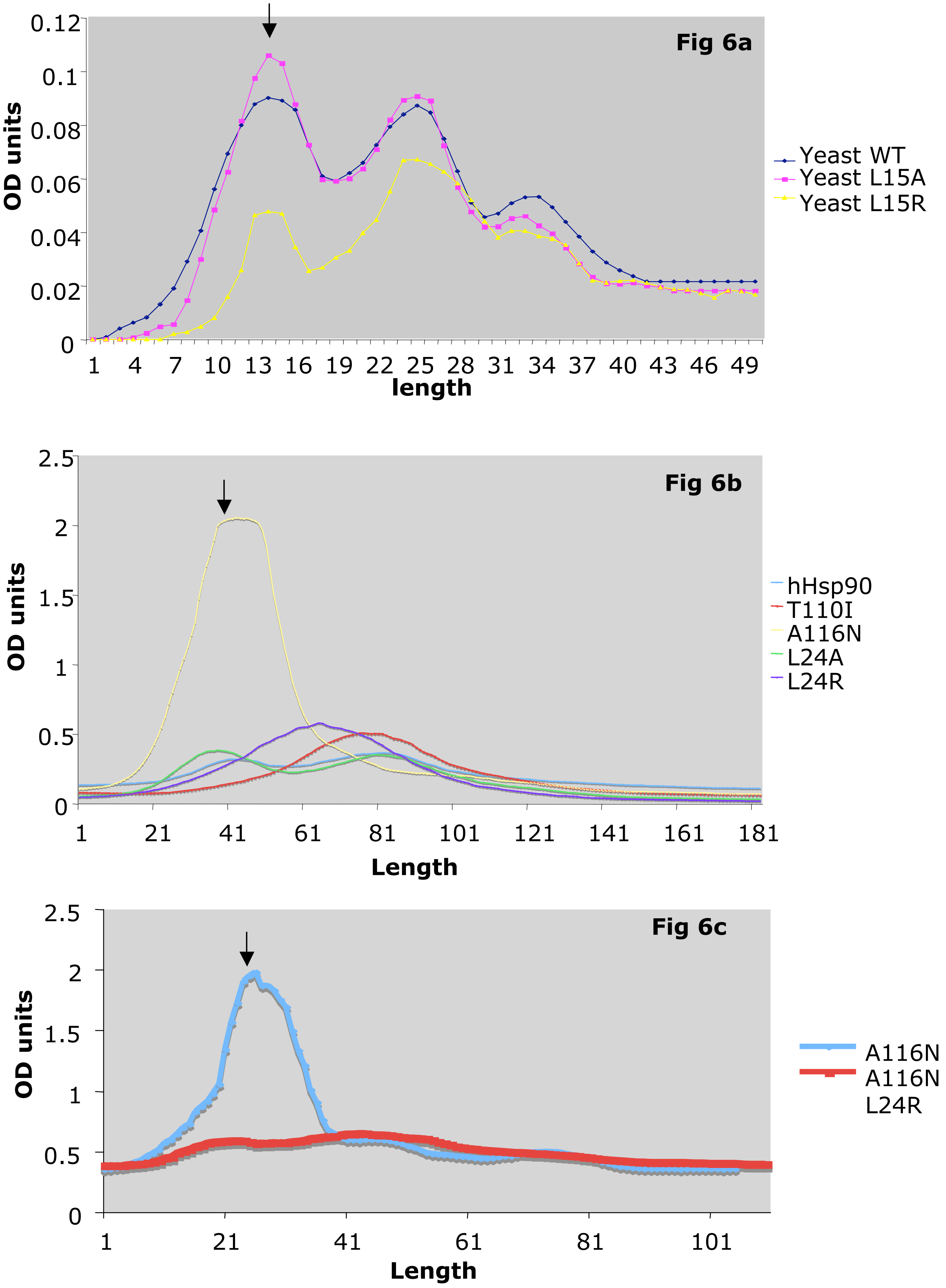

Supplementary figure 2. Gels were scanned and were necessary normalized against total intensity. The peak representing the slowest migrating band for the yeast Hsp90 is shown by the black arrow.
